# CONTROVERSIES AND CHALLENGES IN DEFINING SARCOPENIC OBESITY

**DOI:** 10.1093/geroni/igz038.1444

**Published:** 2019-11-08

**Authors:** John A Batsis

**Affiliations:** Geisel School of Medicine at Dartmouth, Lebanon, New Hampshire, United States

## Abstract

Sarcopenia is defined as the loss of muscle mass, strength and physical function with aging and in conjunction with obesity, leads to incrementally adverse outcomes. There is no current consensus for defining this disease entity, making an accurate evaluation challenging in both the research and clinical settings. We will review the definitions put forth by the Sarcopenia Definition and Outcomes Consortium and the European Working Group for the Study of Sarcopenia. We will present data highlighting the different diagnostic criteria of sarcopenic obesity and the diagnostic accuracy of common anthropometric measures in measuring adiposity using data from the National Health and Nutrition Examination surveys. The advantages and disadvantages of the different modalities of assessing body composition will be discussed, including body impedance analysis, dual energy x-ray absorptiometry, computer tomography and magnetic resonance imaging in addition to simple, novel clinical screening tools for obesity sarcopenia will be presented.

